# Effects of Osteopathic Manual Therapy on Plantar Pressure Distribution in Adolescents with Functional Lower Limb Postural Deviations: A Pilot Study

**DOI:** 10.3390/children13101399

**Published:** 2026-10-14

**Authors:** Mihai-Alexandru Cîtea, Paul Lucaci, Marius Neculăeș

**Affiliations:** Faculty of Physical Education and Sports, “Alexandru Ioan Cuza” University of Iași, 3 Toma Cozma Street, 700554 Iasi, Romania; citea.alexandru@uaic.ro (M.-A.C.); marius.neculaes@uaic.ro (M.N.)

**Keywords:** osteopathy, plantar arch, lower limb malalignment, prevention

## Abstract

**Highlights:**

**What are the main findings?**

• Osteopathic manual therapy was followed by a significant redistribution of plantar loading from the rearfoot toward the forefoot.
• A significant change in the right dynamic Arch Index was observed, whereas mediolateral plantar-load distribution remained largely unchanged.

**What are the implications of the main findings?**

• Instrumented plantar-pressure assessment may help identify short-term functional changes in foot-loading patterns among adolescents with postural deviations.
• These preliminary findings provide a basis for further controlled studies investigating the potential role of osteopathic manual therapy in managing functional postural deviations.

**Abstract:**

(1) Background: Adolescence is a period of rapid musculoskeletal development during which functional postural deviations may occur without structural deformity. This pilot study investigated changes in the plantar pressure distribution following osteopathic manual therapy (OMT) in adolescents with functional postural deviations. (2) Materials and Methods: Twenty adolescents aged 14–16 years (8 females and 12 males) participated in this study. Functional postural status was characterized using clinical screening and Kineod three-dimensional postural assessment, while plantar pressure outcomes were recorded using the BTS P-WALK platform under static and dynamic conditions. Participants completed 10 individualized OMT sessions over three months, consisting primarily of soft-tissue and muscle-energy techniques. (3) Results: Mediolateral plantar load distribution did not change significantly. Forefoot loading increased from 32.65 ± 12.58% to 39.67 ± 9.29%, with a reciprocal reduction in rearfoot loading from 67.35 ± 12.58% to 60.34 ± 9.29% (Holm-adjusted *p* = 0.006; Cohen’s *dz* = 0.842). The right dynamic Arch Index also increased significantly from 13.73 ± 8.86% to 17.37 ± 7.44% (Holm-adjusted *p* = 0.025; *dz* = 0.701). Other Arch Index changes were not significant after correction for multiple testing. (4) Conclusions: In this pilot cohort, OMT was followed by measurable changes in the forefoot–rearfoot plantar load distribution and the right dynamic Arch Index. These findings should be interpreted as preliminary functional changes in plantar loading rather than evidence of structural correction or definitive therapeutic efficacy. Larger controlled studies are needed to determine whether these changes are specifically attributable to OMT.

## 1. Introduction

Adolescence is a critical period for musculoskeletal development, during which postural deviations may become clinically relevant and may be associated with musculoskeletal complaints [1]. Early identification of such deviations and the use of conservative interventions have therefore received increasing attention, particularly as exercise-based approaches have shown potential to improve postural parameters and musculoskeletal outcomes in young populations [2,3,4].

Maintaining good posture requires a well-coordinated and harmonious interaction between the joints, bones, muscles, and nervous system. Proper movement mechanics also play a crucial role in supporting posture [5,6]. Both static and dynamic postural responses are flexible and automatic, adapting to external stimuli, movement, injury, or physical effort. These responses reflect how individuals physically interact with their surroundings [5,6]. The way body weight is distributed while standing results from the intricate coordination of several systems, such as proprioceptive feedback, motor control, kinesthetic memory, and adequate joint mobility [5,6].

The sacroiliac joint (SIJ) contributes to load transfer between the axial skeleton, pelvis, and lower limbs and therefore forms part of the lumbopelvic biomechanical chain during standing and locomotion [7]. However, the extent to which changes in SIJ function translate into measurable alterations in distal plantar loading remains uncertain, and a direct causal relationship should not be assumed.

Experimental studies have examined whether SIJ manipulation is followed by changes in plantar loading measures. Grassi et al. [8] reported changes in between-foot plantar pressure distribution following SIJ manipulation in asymptomatic individuals, while Méndez-Sánchez et al. [9], in a randomized controlled double-blind study, identified immediate between-group differences in selected plantar load variables following bilateral SIJ manipulation. Conversely, Guimarães et al. [10] reported an association between SIJ mobility and standing center-of-gravity measures but did not identify significant associations with plantar pressure or plantar contact area.

Anker et al. (2008) [11] examined the relationship between weight-bearing asymmetry and postural stability in healthy individuals. Greater asymmetry in weight distribution was associated with increased postural sway velocity, particularly in the mediolateral direction, suggesting that unequal loading between the lower limbs may be related to reduced postural control [11].

Within this biomechanical and sensorimotor context, functional deviations involving the spine, pelvis, or hip region may influence both static and dynamic postural control through changes in proprioceptive input, neuromuscular coordination, and joint mobility [5,6].

Manual therapy comprises a range of non-invasive, hands-on interventions used in the management of musculoskeletal dysfunctions. Depending on the technique and clinical context, manual therapy may contribute to improvements in pain, joint mobility, and soft-tissue function [12,13].

In a review focused on adolescent idiopathic scoliosis, Lotan and Kalichman reported that manual therapy approaches, including manipulation, mobilization, and soft-tissue techniques, were associated with improvements in selected post-treatment outcomes, although the available evidence remained heterogeneous [14].

The medial longitudinal arch contributes to load distribution and functional stability of the foot during standing and locomotion. Variations in arch morphology, including reduced arch height, have been associated with altered plantar pressure patterns and postural control characteristics in children and adolescents [15].

Despite these findings, the heterogeneity of manual therapy techniques and the multifactorial nature of postural control make it difficult to compare results across studies and to identify intervention-specific effects. Further research using objective biomechanical outcomes is therefore warranted to clarify the potential influence of manual therapy on plantar load distribution and related postural parameters.

Evidence regarding changes in plantar load distribution following manual therapy in adolescents remains limited, particularly in individuals presenting functional postural deviations without clinically evident structural musculoskeletal deformities. Investigating such changes may provide preliminary information on the relationship between manual therapy and plantar loading patterns in this population and may help inform the design of subsequent controlled studies.

Quantitative assessment of spinal curvature and postural alignment may also provide relevant information regarding biomechanical stability, as measurable inter-individual differences in spinal posture have been reported and should be considered when characterizing postural function [16].

Accordingly, the aim of this pilot study was to investigate changes in the plantar pressure distribution and Arch Index following a three-month individualized OMT program in adolescents presenting asymptomatic functional lower-limb postural deviations without clinically evident structural musculoskeletal deformities.

We hypothesized that OMT would be associated with measurable changes in plantar load distribution and selected Arch Index parameters. Given the exploratory pilot design, this study was intended to provide preliminary estimates and to generate hypotheses for subsequent controlled investigations rather than to establish definitive therapeutic efficacy.

## 2. Materials and Methods

### 2.1. Study Participants

This study was designed as a prospective, single-arm exploratory pilot intervention study examining pre- to post-intervention changes in plantar pressure parameters following an individualized osteopathic manual therapy program.

This study included 20 high school students aged 14–16 years, comprising 8 females and 12 males. At recruitment, none of the participants presented clinically evident structural musculoskeletal deformities or previously diagnosed orthopedic conditions.

However, standardized clinical screening and instrumented assessment identified functional postural deviations in all participants. Clinical screening was performed by the same physiotherapist who delivered the intervention. The clinician had more than 10 years of professional experience and had completed structured postgraduate training in osteopathy. For the purposes of this exploratory pilot study, a functional postural deviation was operationally defined as the presence of at least 10 measured parameters outside the prespecified system-defined reference values used within the clinical and instrumented assessment battery, in the absence of clinically evident structural musculoskeletal deformity.

The threshold of at least 10 parameters outside the system-defined reference values was used as an operational study-specific eligibility criterion and should not be interpreted as a validated diagnostic cutoff or as evidence of structural orthopedic pathology.

These deviations were interpreted as functional assessment findings rather than as evidence of structural orthopedic pathology. Instrument-based reference ranges were those implemented within the Kineod (AXS Medical SAS, Mauguio, France) assessment system and were used solely to identify departures from the system-defined reference values, not to establish a structural orthopedic diagnosis.

Prior to enrollment, written informed consent was obtained from the participants and their legal guardians. All study procedures were conducted in accordance with the Declaration of Helsinki and its subsequent amendments.

The inclusion criteria were: (1) age between 14 and 16 years; (2) absence of visible or documented structural musculoskeletal deformities; (3) presence of asymptomatic functional postural deviations, defined by at least 10 assessed parameters outside the system-defined reference values; (4) no previous participation in postural rehabilitation or therapeutic exercise programs; (5) willingness to complete the study protocol; and (6) provision of written informed consent by the participant and legal guardian.

The exclusion criteria were: (1) the presence of a visible, structural, or congenital musculoskeletal deformity; and (2) previous participation in a therapeutic program specifically intended to modify postural alignment.

### 2.2. Assessment Tool and Procedure

Clinical screening was performed primarily to identify visible musculoskeletal abnormalities or previously documented structural conditions that would preclude inclusion in the functional pilot cohort. Instrumented assessment was subsequently performed using the Kineod (AXS Medical SAS, Mauguio, France) three-dimensional postural analysis system and the BTS P-WALK (BTS S.p.A., Garbagnate Milanese, Italy) plantar pressure platform.

#### 2.2.1. Kineod Postural Assessment

The Kineod system was used for non-ionizing three-dimensional postural assessment. Before acquisition, the following anatomical landmarks were identified and marked: the C7 spinous process, inferior angles of the scapulae, posterior superior iliac spines, the S1 level of the median sacral crest, popliteal fossae, Achilles tendon insertions on the calcanei, and palpable vertebral spinous processes. Participants were assessed in a natural standing position at a distance of approximately 1.5–1.7 m from the device. Following validation of the body contour and anatomical landmarks, image acquisition was performed over approximately 33 ms.

The following Kineod parameters were assessed: thoracic kyphosis angle, lumbar lordosis angle, relative height of the inflection point, shoulder imbalance, pelvic imbalance, scapular imbalance, knee imbalance, left knee deviation, right knee deviation, malleolar imbalance, C7 deviation from the Barré vertical, pelvic deviation from the Barré vertical, and the Kineod angle, defined as the angular magnitude of a frontal-plane spinal curvature identified from the reconstructed spinal line.

These measurements were obtained at both the baseline and post-intervention assessment. Kineod parameters were used to characterize functional postural status and its evolution over the study period; however, they were not designated as primary outcomes in the present analysis, which focused on plantar pressure distribution and the Arch Index.

#### 2.2.2. P-WALK Evaluation

Previous research using the P-WALK platform has reported good repeatability for selected static plantar pressure and load distribution parameters [17].

Plantar pressure data were acquired using a single-plate P-WALK baropodometric platform (BTS S.p.A., Garbagnate Milanese, Italy). The platform measured 675 × 540 × 5 mm and incorporated an active sensing area of 480 × 480 mm comprising 2304 resistive sensors, each measuring 10 × 10 mm. The system operated within a pressure range of 30–400 kPa and supported acquisition frequencies of up to 100 Hz. Data acquisition, processing, and report generation were performed using G-Studio software (BTS Bioengineering, Garbagnate Milanese, Italy, version 3.3.22.0).

For the static assessment, participants stood barefoot on the designated markings at the center of the platform in a relaxed bipedal stance, with their gaze directed toward a fixed point ahead. They were instructed to remain as still as possible for 30 s, during which a 20 s data acquisition period was recorded.

For the dynamic assessment, participants walked barefoot at a comfortable, self-selected pace along an approximately 8 m walkway, with the P-WALK platform positioned centrally. Walking trials were repeated until at least five valid complete foot contacts had been recorded for each foot. Trials in which the foot did not make complete contact with the sensing area were excluded from the analysis.

During the static assessment, the following P-WALK parameters were recorded: left-foot load, right-foot load, forefoot load, rearfoot load, left static Arch Index, and right static Arch Index. During the dynamic assessment, the left and right dynamic Arch Index values were recorded.

According to the classification implemented in G-Studio, the Arch Index values were categorized as follows: 0–7%, Heavy High Arch; 7–14%, High Arch; 14–21%, Light High Arch; 21–28%, Normal Foot; 28–35%, Light Flat Foot; 35–42%, Flat Foot; and 42–100%, Heavy Flat Foot.

Four additional dynamic P-WALK variables were recorded during the broader assessment but were not included among the primary intervention outcomes: left step contact duration, right step contact duration, left foot contact area, and right foot contact area.

**Figure 1:**
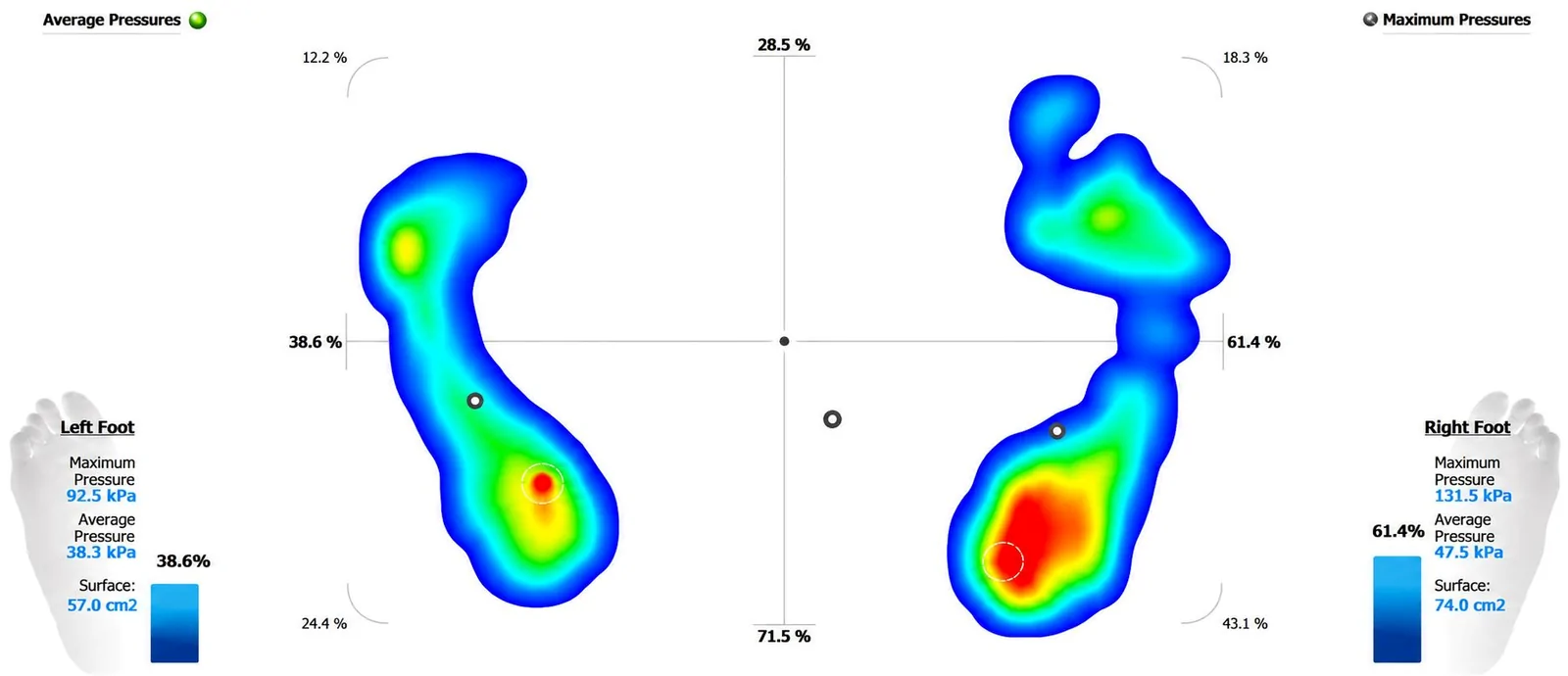
G-Studio report—static plantar pressure evaluation.

Data were processed and reported using the G-Studio software (Figure 1).

The primary outcomes of interest were the plantar load distribution and Arch Index parameters, assessed under static and dynamic conditions as described above.

**Figure 2:**
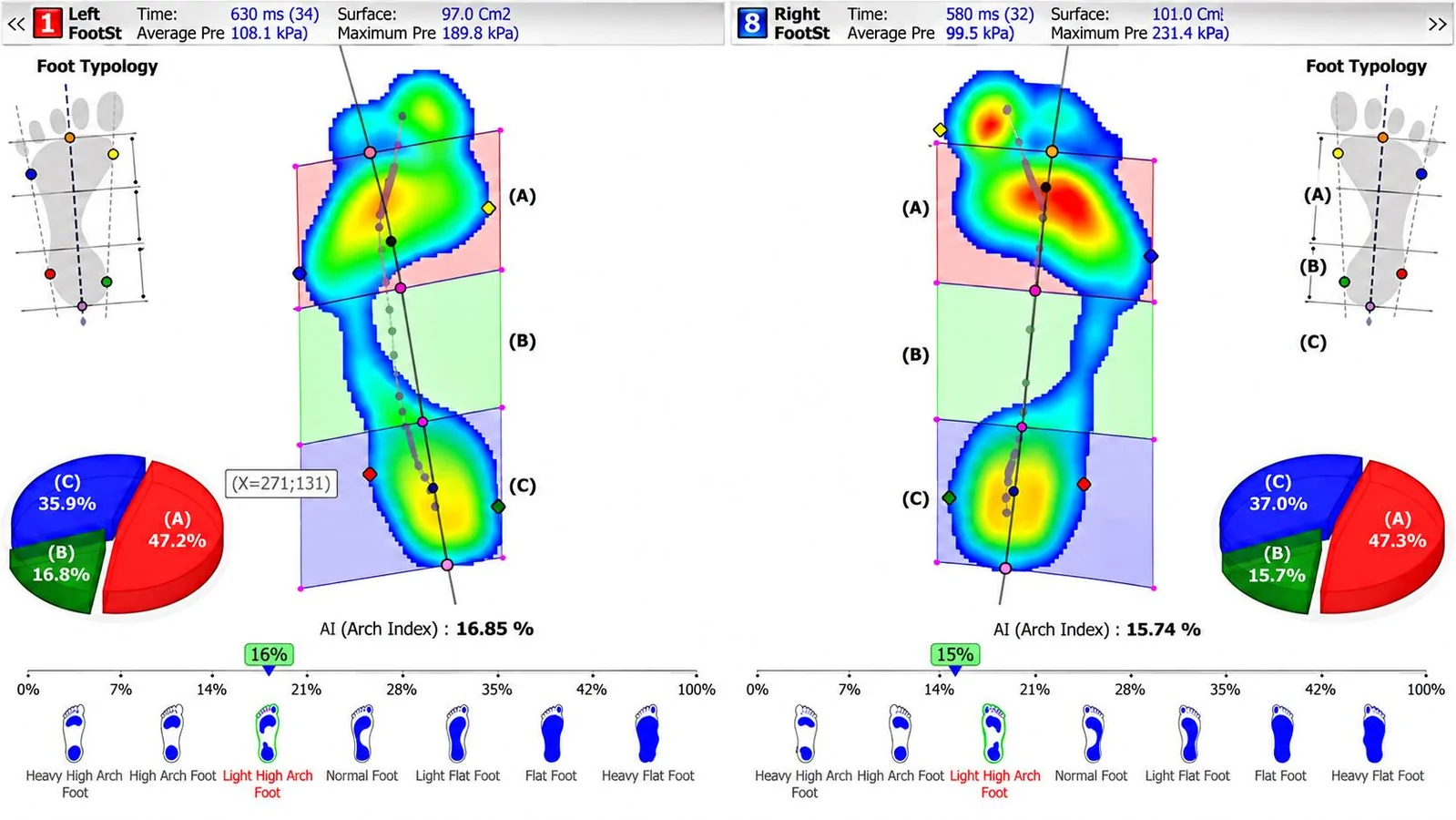
G-Walk report—dynamic plantar pressure evaluation. The plantar surface is divided into three regions: (A) forefoot, (B) midfoot, and (C) rearfoot (hindfoot).

The dynamic evaluation involved having each participant walk naturally across a room with a minimum length of 8 m. The BTS P-WALK pressure plate was positioned at the center of the room to capture gait data as the participants moved from one end to the other. Each subject was instructed to walk at a comfortable, self-selected pace and to complete at least 10 laps to ensure sufficient data collection (Figure 2).

During the assessment, the evaluator monitored each pass to confirm that the participant’s foot made full contact with the pressure plate. Any steps that were incomplete or partially recorded (e.g., heel or toe off the platform) were excluded from the analysis. Once the software recorded at least five valid steps for each foot, it generated a dynamic report of various gait and plantar pressure parameters.

### 2.3. Intervention Details

The intervention comprised osteopathic manual techniques grouped into two predefined categories: soft-tissue/myofascial techniques and muscle-energy techniques. Treatment was individualized according to the functional findings identified during assessment and targeted the spinal, thoracolumbar, rib, pelvic, sacroiliac, and hip regions. Soft-tissue techniques involved low-to-moderate pressure applied through longitudinal or transverse tissue loading.

Each treatment session lasted approximately 60 min and was administered once weekly. All participants completed the full 10-session intervention protocol. No adverse events were reported during the intervention period. The selection, sequencing, and progression of manual techniques were individualized according to the tissue response observed during treatment, including changes in perceived tissue tension and resistance.

The intervention included osteopathic soft-tissue and myofascial techniques, such as seated thoracic paravertebral soft-tissue release with thoracic extension, prone transverse lumbar paravertebral soft-tissue release, lumbosacral paravertebral separation and myofascial release, bilateral lumbar paravertebral thumb stretching, lumbar paravertebral release with hip extension and adduction, lumbar paravertebral release with pelvic elevation, side-lying and supine lumbar paravertebral soft-tissue release, supine thoracolumbar fascial release with lower-body rotation, seated lumbar paravertebral release with trunk flexion and rotation, and levator scapulae myofascial release. Muscle-energy techniques were applied to address rib mobility dysfunction and functional restrictions involving the quadratus lumborum, bilateral hip adductors and abductors, iliopsoas, and piriformis. Unilateral and bilateral sacral extension/counternutation and flexion/nutation techniques assisted by respiration were also included.

All OMT sessions were delivered by the same physiotherapist, who had more than 10 years of clinical experience and had completed a structured osteopathy training program provided by the Romanian Society of Osteopathy between 2015 and 2021, together with additional postgraduate training in orthopedic manual therapy.

Techniques were selected from the predefined treatment repertoire according to the participant’s functional findings and tissue response. The sequence and number of techniques applied during a session were not fixed; treatment was reassessed during the session according to perceived tissue tension and resistance, and the subsequent technique was selected accordingly.

Soft-tissue applications were generally maintained for 20–120 s per treated segment or until a perceptible reduction in tissue tension was achieved. Muscle-energy techniques involved positioning the treated segment at the first restrictive barrier, followed by a voluntary isometric contraction against graded resistance, relaxation, and passive repositioning toward the new barrier. Isometric contractions were generally maintained for 3–5 s and repeated three to seven times, whereas respiration-assisted sacral techniques were performed over approximately five to ten respiratory cycles.

### 2.4. Statistical Analysis

Statistical analyses were performed using SPSS version 29.0.1.0. Descriptive statistics were calculated for each outcome, including the mean, standard deviation, median, interquartile range, minimum, and maximum values.

Pre- to post-intervention differences were evaluated using paired-samples *t*-tests. For paired comparisons, effect sizes were expressed as Cohen’s *dz*, with values of approximately 0.2, 0.5, and 0.8 interpreted as small, moderate, and large effects, respectively.

Because left- versus right-foot load and forefoot versus rearfoot load represent complementary percentage distributions, each pair was considered a single non-redundant outcome for multiplicity control.

To account for multiple comparisons across the six non-redundant outcomes, *p*-values were adjusted using the Holm procedure. Statistical significance was set at an adjusted *p* < 0.05.

The distribution of the within-participant paired differences was assessed for approximate normality before application of the paired-samples *t*-tests. The six non-redundant outcomes included in the Holm family were: mediolateral plantar load distribution, anteroposterior plantar load distribution, left static Arch Index, right static Arch Index, left dynamic Arch Index, and right dynamic Arch Index.

## 3. Results

Eight plantar pressure and Arch Index parameters were analyzed to characterize pre- to post-intervention changes. All 20 participants completed the post-intervention assessment, and their data were included in the statistical analysis.

The analysis included the following eight variables: left-foot load, right-foot load, forefoot load, rearfoot load, left and right static Arch Index, and left and right dynamic Arch Index. These variables were used to characterize the plantar load distribution and Arch Index under static and dynamic conditions.

Left- and right-foot load were expressed as percentages of total plantar load, with 50% per limb representing an equal bilateral distribution. This value was used as a descriptive reference for symmetry rather than as a diagnostic threshold.

The Arch Index values were interpreted according to the seven-category classification implemented in G-Studio, as described in the Methods section. These software-defined categories were used descriptively to characterize plantar-arch contact patterns and were not interpreted as diagnostic categories of structural or pediatric flatfoot. Pre- to post-intervention changes in plantar load distribution are summarized in Table 1.

Mediolateral plantar load distribution showed only a small pre- to post-intervention change. Left-foot load increased by 0.86 percentage points, with a reciprocal decrease of 0.86 percentage points in right-foot load. This complementary redistribution was not statistically significant after Holm correction (adjusted *p* = 0.846; Cohen’s *dz* = 0.248).

In contrast, anteroposterior plantar load distribution showed a larger change. Forefoot load increased from 32.65% to 39.67%, while rearfoot load decreased reciprocally from 67.35% to 60.34%, corresponding to a redistribution of approximately 7.02 percentage points. This change remained statistically significant after correction for multiple comparisons (Holm-adjusted *p* = 0.006) and was associated with a large within-participant effect size (Cohen’s *dz* = 0.842). Pre- to post-intervention changes in static and dynamic Arch Index are summarized in Table 2.

The Arch Index changes were not uniform across sides or assessment conditions. The left static Arch Index increased from 6.68% to 9.27%. Although the nominal *p*-value was 0.035, this difference did not remain statistically significant after Holm correction (adjusted *p* = 0.140; Cohen’s *dz* = 0.508). Changes in the right static and left dynamic Arch Index were small and were not statistically significant after multiplicity correction.

The right dynamic Arch Index showed the largest Arch Index change, increasing from 13.73% to 17.37%, corresponding to a mean increase of 3.64 percentage points. This difference remained statistically significant after Holm correction (adjusted *p* = 0.025; Cohen’s *dz* = 0.701). Descriptively, this finding reflects increased midfoot plantar contact during dynamic assessment; however, its clinical or structural significance cannot be inferred from the Arch Index change alone.

Overall, two of the six non-redundant outcome comparisons remained statistically significant after Holm correction: anteroposterior forefoot–rearfoot load redistribution and right dynamic Arch Index. The remaining outcomes did not retain statistical significance after adjustment for multiple comparisons.

## 4. Discussion

Demirbüken et al. (2019) identified early adolescence as a period during which plantar pressure distribution may undergo age- and sex-related changes, highlighting the importance of age-appropriate interpretation of plantar loading characteristics [18]. Within this developmental context, objective assessment of plantar pressure distribution may provide useful information regarding functional adaptations that are not readily identified through conventional postural observation alone.

The present pilot study investigated changes in the plantar load distribution and Arch Index following a three-month individualized OMT program in adolescents with functional postural deviations. The findings demonstrate that measurable changes occurred in selected plantar pressure parameters, with the clearest responses involving the anteroposterior plantar load distribution and right dynamic Arch Index. Importantly, both outcomes remained statistically significant after adjustment for multiple comparisons.

Manual therapy has previously been investigated in a range of musculoskeletal conditions, including inversion ankle sprain, plantar heel pain, plantar fasciitis, and pediatric populations [19,20,21,22]. However, differences in clinical populations, treatment techniques, and outcome measures limit direct comparison with the present study.

The observed changes in plantar load distribution followed a treatment program based primarily on soft-tissue techniques and muscle-energy techniques. Previous research has reported that soft-tissue mobilization may influence musculoskeletal properties and joint range of motion [23], while muscle-energy techniques have been investigated for their effects on pain and range of motion in both symptomatic and asymptomatic populations [13]. These previously described effects may provide a plausible biomechanical context for the present findings.

The mediolateral plantar load distribution showed a small descriptive change following the intervention. Mean left-foot load increased by 0.86 percentage points, with a reciprocal decrease of 0.86 percentage points in the right-foot load. Because the left- and right-foot loads are complementary percentages, these changes represent a single mediolateral redistribution. The change was not statistically significant after Holm correction (adjusted *p* = 0.846; Cohen’s *dz* = 0.248), indicating that the mediolateral load distribution remained relatively stable across the intervention period.

In contrast to the mediolateral findings, the anteroposterior plantar load distribution showed a larger pre- to post-intervention change. Mean forefoot load increased from 32.65% to 39.67%, with a reciprocal decrease in rearfoot load from 67.35% to 60.34%, corresponding to a redistribution of 7.015 percentage points. This change remained statistically significant after correction for multiple comparisons (Holm-adjusted *p* = 0.006) and was associated with a large within-participant effect size (Cohen’s *dz* = 0.842). Previous studies have reported variability in the anteroposterior plantar load distribution across different populations, ranging from approximately balanced forefoot–rearfoot loading to relatively greater rearfoot loading [24,25]. Recent clinical research has also used baropodometric outcomes to examine changes in plantar pressure distribution following manual therapy. Falaguera-Vera et al. [26] reported changes in dynamic plantar pressure characteristics following a manual therapy program.

A reduction in dispersion was also observed at the post-intervention assessment. For forefoot and rearfoot load, the variance decreased from 158.16 to 87.85, while for left- and right-foot load, it decreased from 36.60 to 22.02. These changes indicate that individual values were less dispersed around the group mean at the post-intervention assessment.

The anteroposterior plantar load redistribution was statistically significant after correction for multiple comparisons (Holm-adjusted *p* = 0.006), whereas the mediolateral redistribution was not (Holm-adjusted *p* = 0.846). These findings indicate that, within the present sample, the pre- to post-intervention change was more pronounced for anteroposterior than for mediolateral plantar load distribution.

Changes in the Arch Index were not uniform across conditions. The left static Arch Index increased from 6.68% to 9.27%, but this difference was not statistically significant after correction for multiple comparisons (Holm-adjusted *p* = 0.140; Cohen’s *dz* = 0.508). In contrast, the right dynamic Arch Index increased from 13.73% to 17.37% and remained statistically significant after Holm correction (adjusted *p* = 0.025; Cohen’s *dz* = 0.701). The right static and left dynamic Arch Index showed only small, non-significant changes. Overall, the findings indicate condition-specific changes in plantar-contact patterns rather than a uniform modification of arch characteristics.

The observed Arch Index changes provide additional evidence that the plantar-contact characteristics were modified over the intervention period. Because structural foot alignment was not assessed using radiographic imaging or conventional orthopedic angular measurements, these findings are best interpreted as functional changes in plantar contact rather than as structural remodeling. Previous studies have reported changes in foot-related outcomes following conservative interventions. In children with flatfoot, Boryczka-Trefler et al. found that the addition of Zukunft-Huber manual therapy to foot orthoses was associated with greater changes in selected plantar-contact and pressure parameters than orthoses alone [27]. Exercise-based interventions have also been associated with improvements in clinical measures of flexible flatfoot, including the navicular drop and medial arch angle [28]. Although these findings provide relevant context, differences in population characteristics, interventions, and outcome measures prevent direct comparison with the present study.

These parallels support the view that non-invasive therapies can produce quantitative enhancements in the Arch Index and associated foot geometry.

The Arch Index values were interpreted according to the G-Studio classification described in the Methods section. Within this software-defined framework, values below 21% fall within one of the high-arch categories, whereas values between 21% and 28% are classified as Normal Foot. Although the mean Arch Index values generally increased after the intervention, none of the group means reached the software-defined Normal Foot range. The most pronounced change was observed for the right dynamic Arch Index, which increased from 13.73% to 17.37%, corresponding descriptively to a shift from the High Arch to the Light High Arch category.

Previous studies indicate that conservative interventions may modify selected functional measures of foot posture and plantar loading. Markowicz et al. reported improvements in balance control following a rehabilitation program targeting the intrinsic foot muscles in children with flat feet [29]. Exercise-based interventions have also been associated with changes in foot-alignment measures in individuals with flexible flatfoot [28], while Boryczka-Trefler et al. reported additional changes in plantar-contact and pressure parameters when Zukunft-Huber manual therapy was combined with foot orthoses in children [27]. These findings provide a broader context in which functional foot-related measures may respond to conservative intervention.

Previous studies have reported associations between foot arch characteristics, plantar loading, and measures of postural control in different populations [29,30,31]. However, the present study did not directly assess changes in postural stability, gait symmetry, tibial rotation, knee alignment, or structural foot geometry. Therefore, the observed Arch Index changes should not be interpreted as evidence of improved balance, normalization of foot posture, or correction of lower-limb alignment.

Within the G-Studio classification, the mean Arch Index values increased during the study period, but none of the group means reached the software-defined Normal Foot range. The right dynamic Arch Index showed the most pronounced and statistically robust change, increasing from 13.73% to 17.37%, corresponding descriptively to a shift from the High Arch to the Light High Arch category. This finding is best interpreted as a change in plantar-contact behavior during dynamic assessment rather than as structural normalization of the medial longitudinal arch.

In summary, the present pilot study identified measurable pre- to post-intervention changes in selected plantar pressure parameters, particularly the anteroposterior load distribution and right dynamic Arch Index. These findings extend the limited literature examining plantar pressure outcomes following manual therapy in younger populations. However, because no control group was included, the observed changes should be regarded as exploratory and cannot be attributed specifically to OMT. Future controlled studies are needed to determine whether these changes exceed natural or measurement-related variation and whether they have clinically meaningful consequences.

Our findings generally support the concept that conservative treatments normalize foot structure [27,28] and specifically show that osteopathic or chiropractic techniques have a similar effect.

To our knowledge, few prior studies have directly measured Arch Index outcomes following manual therapy in youths. The current results suggest that integrating manual alignment techniques may accelerate the restoration of normal foot geometry in adolescents with postural deviations. Future work should compare manual therapy head-to-head with other modalities and evaluate the durability of these corrections, but the present data indicate that manual therapy is a viable strategy to improve plantar arch posture and related biomechanical health.

An increased Arch Index indicates greater midfoot plantar contact; however, the direction of this change should not be interpreted as inherently beneficial in the absence of independent structural assessment and clinical context.

This study presented a set of limits that must be taken into account. The specific set of manual therapy used has a global effect, and as such, we cannot tell with precision if the outcome shown is the result of all of the interventions or of a specific element performed on one or more areas. This makes further studies necessary to evaluate the short- and long-term effect of different techniques. At the same time, the low number of participants may offer only a vague glimpse into the specific pattern of weight distribution at the plantar level for 14–16-year-old highschoolers, and it would be more beneficial to have a wider range for a clear conclusion to be drawn.

Although these changes were statistically significant, no established minimal clinically important difference is available for these measures in asymptomatic adolescents. Their clinical and biomechanical significance therefore remain uncertain.

The isolated change observed in the right dynamic Arch Index should be interpreted cautiously. Among the four static and dynamic Arch Index measurements, only the right dynamic value remained statistically significant after multiplicity correction. The present data do not allow a mechanistic explanation for this side-specific response. Potential contributors may include baseline inter-limb asymmetry, habitual loading patterns, lower-limb dominance, individual biological variability, or variability related to dynamic testing; however, these factors were not specifically examined in the present study. The finding should therefore be regarded as exploratory and require replication in a controlled study before any side-specific mechanism can be proposed.

## 5. Conclusions

In this pilot cohort of adolescents with functional lower-limb postural deviations, a three-month individualized OMT program was followed by measurable changes in selected plantar pressure parameters.

The clearest change was observed in the anteroposterior plantar load distribution, with forefoot load increasing from 32.65% to 39.67% and rearfoot load decreasing reciprocally from 67.35% to 60.34%. This redistribution of 7.015 percentage points remained statistically significant after correction for multiple comparisons (Holm-adjusted *p* = 0.006; Cohen’s *dz* = 0.842). The right dynamic Arch Index also increased significantly after correction (Holm-adjusted *p* = 0.025; Cohen’s *dz* = 0.701). Although the left static Arch Index showed a nominal *p*-value of 0.035, this change did not remain statistically significant after Holm correction (adjusted *p* = 0.140).

Taken together, these preliminary findings suggest that OMT may be associated with short-term changes in selected plantar loading parameters in adolescents with functional postural deviations. Larger controlled studies are required to determine whether these changes can be attributed specifically to the intervention and whether they are clinically meaningful and sustained over time. By promoting structural balance and more physiologic plantar loading patterns, such interventions may reduce future risk of musculoskeletal dysfunction. Further research with larger samples, extended follow-up, and comparative modalities is warranted to consolidate these findings and determine long-term outcomes.

## 6. Limitations

This study included a relatively small sample of 20 adolescents. This limited sample size reduces statistical precision and restricts the generalizability of the findings to broader adolescent populations. Accordingly, the present investigation should be regarded as a pilot study providing preliminary estimates of changes in plantar pressure outcomes following OMT rather than definitive evidence of therapeutic efficacy.

A major limitation is the absence of a control group. Consequently, the pre- to post-intervention changes cannot be attributed specifically to OMT. Natural maturation, repeated testing and familiarization, day-to-day biological variability, and regression to the mean may have contributed to the observed differences. Regression to the mean is of particular relevance because eligibility required at least 10 assessment parameters to fall outside the system-defined reference values at baseline. This selection criterion may increase the probability that some relatively extreme baseline measurements move closer to typical values on repeat assessment independently of a true intervention effect.

The osteopathic intervention was individualized and included a combination of several manual techniques, in keeping with usual clinical practice. This approach increases the clinical relevance of the intervention but does not allow the specific contribution of each individual technique to be determined.

This study focused on short-term changes and did not include a longer post-intervention follow-up. Therefore, future studies would be useful to determine whether the observed plantar pressure adaptations are maintained over time.

Walking speed was self-selected and was not instrumentally recorded or standardized between assessments; therefore, differences in walking velocity may have contributed to variability in the dynamic plantar pressure measures.

Because the participants were asymptomatic, outcomes such as pain, disability, quality of life, and injury incidence were not central to the present investigation. Accordingly, the present findings primarily reflect biomechanical and postural changes, and their broader clinical relevance should be explored in future research.

## Figures and Tables

**Table 1:** Pre- to post-intervention changes in plantar load distribution (n = 20).

Outcome	Baseline, Mean ± SD (%)	Post-Intervention, Mean ± SD (%)	Mean Change (pp)	95% CI for Mean Change	Nominal *p*	Holm-Adjusted *p*	Cohen’s *dz*
Left-foot load	42.52 ± 6.05	43.38 ± 4.69	0.86	−0.77 to 2.49	0.282	0.846	0.248
Right-foot load	57.49 ± 6.05	56.63 ± 4.69	−0.86	−2.49 to 0.77	0.282	0.846	0.248
Forefoot load	32.65 ± 12.58	39.67 ± 9.29	7.02	3.12 to 10.91	0.001	0.006	0.842
Rearfoot load	67.35 ± 12.58	60.34 ± 9.29	−7.02	−10.91 to −3.12	0.001	0.006	0.842

**Table 2:** Pre- to post-intervention changes in static and dynamic Arch Index (n = 20).

Outcome	Baseline, Mean ± SD (%)	Post-Intervention, Mean ± SD (%)	Mean Change (pp)	95% CI for Mean Change	Nominal *p*	Holm-Adjusted *p*	Cohen’s *dz*
Left static Arch Index	6.68 ± 7.97	9.27 ± 7.68	2.59	0.21 to 4.98	0.035	0.14	0.508
Right static Arch Index	16.95 ± 8.36	17.14 ± 8.10	0.18	−0.86 to 1.22	0.72	0.846	0.081
Left dynamic Arch Index	11.41 ± 8.67	12.12 ± 8.44	0.71	−0.67 to 2.08	0.295	0.846	0.241
Right dynamic Arch Index	13.73 ± 8.86	17.37 ± 7.44	3.64	1.21 to 6.08	0.005	0.025	0.701

## Data Availability

The original contributions presented in this study are included in the article. Further inquiries can be directed to the corresponding author.
